# Supplementary material for: You can’t report your feelings: The hidden labor of managing threats to safety by women in global public health fieldwork
Source: PLOS Glob Public Health. 2022 Jun 27;2(6):e0000153. doi: 10.1371/journal.pgph.0000153 (PMC10022030; doi:10.1371/journal.pgph.0000153)
Supplement: S1 Text — (PDF) [file pgph.0000153.s001.pdf]

In-depth Interview Guide

Main research question:

What was it like to practice global health abroad during your graduate public health training?

Prompts used in the initial interview focus on capturing and eliciting lived experience. Examples of these types of questions include:

- *Please describe an experience of global public health practice in as much detail as possible.*
- *Will you walk me through that day?*
- *Tell me what happened, step by step.*
- *Can you tell me an experience of when you felt that way?*
- *What do you remember?*
- *Tell me a little bit about a typical day when you were in XXX.*
- *Tell me a little bit about how you physically felt. Forget about the feelings but how you physically felt.*

If not addressed spontaneously, further focus on these topics:

- The lived experience of the North American female graduate student participating in global public health practice.
- The difficulties, challenges, distresses, and traumas that female graduate students experience while in the practice of global public health.
- The experience of losing or obtaining support systems and resources.
- The effect on female students' future research, practice, and career orientation.

Do you have anything you would like to add, that you feel you haven’t been able to share during the interview?

Existential Prompts

- Try to think of a particular moment of your global public health practice
- How, where, when did it occur?
- What did you say/do? How did you say it/do it? Tone? Body language?

- What did other people say/do? How did they say it/do it? Tone? Body language?
- What did you notice about other people?
- How did you experience the presence or absence of other people?
- How did the experience feel? What did you notice? What did you not notice?
- How did you experience your surroundings? Or did you?
- Were there specific objects of importance?
- How long did the experience last? How were you aware of time?
- Describe this experience like a state of mind, mood, feelings, emotions
- Do not attempt to clean up your account; rather aim for a direct description of the experience as you lived through it

Additional suggestions for participants:

- Try to avoid abstraction (generalizations, interpretations, rationalization)
- You do not need a follow a story arc (e.g., beginning, middle, and end)
- Focus on an example of the experience which vividly stands out in your memory
- Think of a particular or single example, event, situation – perhaps the first time it happened or the most recent experience
- If appropriate, start your writing with a bodily experience (how the body feels, how things felt, sounded, etc.)
- It may be easier to recall the experience by talking about it in the present tense, rather than in past tense.

Reflective Questions for the Interviewer

What preunderstandings are you bringing to the research process?

How are your understandings fusing with those of the participants' texts and other literature?

Where are your emotional responses signaling what matters to you?

What are you not seeing or responding to?

Are certain interpretations being privileged over others?
