# Supplementary material for: You can’t report your feelings: The hidden labor of managing threats to safety by women in global public health fieldwork
Source: PLOS Glob Public Health. 2022 Jun 27;2(6):e0000153. doi: 10.1371/journal.pgph.0000153 (PMC10022030; doi:10.1371/journal.pgph.0000153)
Supplement: Attachment — Submitted filename: Responses to Reviewers.docx [file pgph.0000153.s002.docx]

| **Reviewer #1 Comments** | **Authors’ Responses** |
| --- | --- |
| Add description of how the stories were developed in the methods section. Where the participants literally writing down aspects, or did the interviewers write the stories and then conduct member checking/have the participants read and make adjustments? It is not clear enough how the stories were really developed. | Thank you for these questions. We have added more detail in the methods section to address what the in-depth phenomenological interviews (IDPI) and the guided writing exercise (GWE) were comprised of (see pg. 8 and 9). To further address your questions, all participants could ask to see their data at any point in the research process. For GWE, I often went back and forth with participants over a few emails to clarify and confirm the accuracy of their story. For IDPI participants, I was able to do this in person during their interviews. Many stories selected for this paper were verbatim (derived from both written and oral data collection), while others that could possibly be identifiable or were repeated in multiple occasions were collated to become a composite based story (see pg. 10). |
| The limitations section should also include a discussion on what/how the researchers' identities may have played in the data collection and analysis, and if phenomenology was the best approach of choice in retrospect: what were benefits and risks by employing it? | Thank you for identifying this need. We have renamed the “Limitations” section, “Strengths and Limitations” and added a paragraph (see pg. 33-34) that addresses the positionality and reflexivity within this study, as well as what phenomenology can and cannot offer regarding these subject matters. |
|  | |
| **Reviewer #2 Comments** |  |
| A brief line providing some background context on the 2018 Workshop on Ethically Managing Global Health Fieldwork Risks. | The following has been added to provide further context (see pg. 4-5), “A 2018 Workshop on Ethically Managing Global Health Fieldwork Risks, co-sponsored by Agnes Scott College, The Taskforce for Global Health, and Emory University Rollins School of Public Health, identified gender-based violence during fieldwork, alongside a culture that then compels silence, as the most concerning theme. Within the academic and global health implementation oriented group, almost every woman present had been affected by gender-based violence, while many men had not realized the severity or extent of the issue (3).” |
| A line or two describing how the participants were recruited and selected would be helpful. | Thank you for this suggestion, we have added the following (see page 8): “We recruited a purposive sample of participants through email and social media, based on the authors’ global health networks. This included reaching out to various Canadian and U.S. schools of public health (e.g., deans, faculty, alumni) and established associations, including student groups connected with these organizations (e.g., Consortium of Universities for Global Health, the now former Canadian Coalition for Global Health Research).” |
| Similarly, a brief description of the two methods of in-depth phenomenological interviews and guided writing exercises would help to make this approach more accessible to readers. | Thank you for this suggestion. We have added more detail in the methods section to address what the in-depth phenomenological interviews (IDPI) and the guided writing exercise (GWE) were comprised of (see pg. 8 and 9). |
| A critique of the larger landscape of global health field work in the global south within which these challenges of hidden labour are embedded would offer a deeper nuance to the discussion. It would be useful to also raise the aspect of accountability on both ends viz. universities in the Global North and host institutions in LMICs. | While a comprehensive critique of the global health field is beyond the scope of this paper and the study upon which it is based, these findings do point to structural barriers facing students pursuing global health studies and calls for greater institutional accountability. We have therefore acknowledged this in the background, discussion, and highlighted the relevance of these findings in this broader landscape and need for further research in this area. Furthermore, we have added the following to attend to the important points brought up by this reviewer (see pg. 32), “Furthermore, accountability is needed on both ends of the spectrum, from universities in the Global North as well as host institutions within LMICs. This necessitates open, productive, reciprocal and equitable conversations and the ability for women to authentically share their experiences as a critical first step in remedying the current situation.” |
